# Can We Implement Multispecialty Mother–Infant Dyadic Care to Systematize Interpregnancy Services After a Preterm Birth?

**DOI:** 10.1089/whr.2023.0148

**Published:** 2023-12-18

**Authors:** Seuli Bose-Brill, Shannon L. Gillespie, Kartik K. Venkatesh

**Affiliations:** ^1^ Combined Internal Medicine and Pediatrics Section, Division of General Internal Medicine, Department of Internal Medicine, The Ohio State University College of Medicine, Columbus, Ohio, USA.; ^2^ The Martha S. Pitzer Center for Women, Children, and Youth, College of Nursing, The Ohio State University, Columbus, Ohio, USA.; ^3^ Division of Maternal Fetal Medicine, Department of Obstetrics and Gynecology, The Ohio State University College of Medicine, Columbus, Ohio, USA.

The United States claims one of the highest rates of pregnancy-related mortality (PRM) and one of the highest rates of infant mortality (IM) among all high-income countries.^1,2^ Such findings are largely driven by the fact that almost one million U.S. pregnant individuals (*i.e.*, 1 in 4) are affected by one or more chronic comorbid conditions, drastically increasing risk for mother and infant.^3–7^ Overall, the higher ages of pregnant individuals combined with a rising maternal chronic disease burden (*e.g.*, hypertension, diabetes, mental health conditions, substance use disorders) and the growing impact of social determinants of health inequities have cumulatively increased the medical and social complexities faced by individuals during and after pregnancy.^1^ Importantly, non-Hispanic Black Americans are also more likely to face a complex pregnancy and/or a new onset complex condition after pregnancy than individuals of any other demographic group, leading to striking racial disparities in maternal and infant health.^8–11^

One complication that is considerably more common among mothers affected by a complex pregnancy is preterm birth (PTB) or birth of an infant before 37 weeks gestation. The United States has one of the highest PTB rates of all countries, affecting 1 in 10 pregnancies.^12^ Again, Black Americans bear a disproportionate burden of the complication, being 1.5 × more likely to have a PTB and more than 2 × more likely to have an early PTB^13^ PTBs (particularly early PTBs) are strong drivers of IM and racial disparities in IM.^14,15^

Mothers transitioning out of a pregnancy affected by PTB are also at higher risk for postpartum morbidity and mortality, new-onset chronic conditions, and a subsequent complicated pregnancy, highlighting the importance of maternal care after a complicated pregnancy. Indeed, longitudinal postpregnancy and interpregnancy clinical care can yield significant public health benefits, including a decreased chronic disease burden among the mother and reduced rates of subsequent PTB.^16–18^ Thus, longitudinal postpartum care is critical for both reducing current and future maternal disease severity and optimizing health for future pregnancies.^19^

Longitudinal postpartum and interpregnancy care serves as a compelling strategy to prevent recurrent adverse pregnancy outcomes, including PTB.^20,21^ For example, the American College of Obstetrics and Gynecology (ACOG) consensus guidelines recommend ongoing, multiepisode longitudinal postpartum and interpregnancy care, personalized to meet individual needs.^16,22^ Per ACOG, optimal interpregnancy care should be delivered by a multidisciplinary team of obstetric, primary care, and subspecialty clinicians, as a continuation of postpartum care to improve short and long-term health outcomes for individuals and their infants.^16^ Furthermore, primary care organizations, such as the Society of General Internal Medicine and the American Association of Family Physicians, have emphasized the importance of postpartum primary care in equitably addressing the health care needs of pregnant individuals across the life course.^23,24^

However, accumulating data has shed light on the inability of current U.S. postpartum health care models to deliver optimal postpartum and interpregnancy care, as the health care infrastructures needed to systematically connect individuals to longitudinal, multidisciplinary care after pregnancy are lacking. Moreover, existing care models often fail to address the needs of minoritized individuals who experience a higher burden of adverse social determinants of health, impeding their access to and utilization of postpartum preventive services.^3,25–29^

As such, few individuals receive any postpartum care and even fewer receive ongoing, longitudinal postpartum and interpregnancy care, even after high-risk pregnancy.^30,31^ Such data are particularly alarming considering that over half of PRM occurs during the year after childbirth,^32^ usually outside of the hospital setting.^32^ Poorly managed chronic conditions during the interpregnancy period also greatly increase risk for complications during subsequent pregnancies, including repeat PTB. Indeed, more than 80% of PRM is considered preventable,^33^ highlighting the significant gap in U.S. maternity care services that occurs during the months and even years after childbirth.^34–36^

Individuals transitioning out of a pregnancy affected by PTB have unique needs. The experience of PTB not only increases risk of subsequent PTB but also results in the need for intensive postpartum health care utilization.^37^ Individuals who experience PTB have a higher risk of postpartum depression, severe maternal morbidity, and all-cause mortality than those who carry pregnancies to term.^38–42^ Yet, they infrequently access primary care in the 12 months after delivery.^43^ Systematizing interpregnancy care after PTB not only has the potential to broadly address poor U.S. maternal health outcomes but also the unacceptable maternal health disparities embedded within these outcomes.^44^ While over 5 decades of research, policy, and practice has informed frameworks and evidence-based practice guidelines for the primary care of the preterm infant during the 1st year and beyond,^45–51^ interpregnancy and postpartum care of the mother after PTB remains a neglected topic.^52–55^

In their article, “Integrating care for mother–infant dyads after preterm birth: A qualitative study of clinician perspectives on feasibility,” Gregory et al. elicit perspectives of multidisciplinary clinicians who provide care to postpartum individuals and infants after PTB to identify potential barriers and facilitators of using pediatric access points for maternal postpartum and interpregnancy care delivery through a dyadic mother–infant care model.^56^ The frequent (often ≥6) pediatric visits in the year after birth has spurred development of focused professional guidelines and reimbursement structures to address maternal health needs for topics such as postpartum depression screening in pediatric settings. However, many unused opportunities remain to leverage infant care access points to address maternal postpartum care gaps.^56^

Some mother/infant dyadic care approaches exist for delivery by providers who care for both postpartum individuals and children, including family medicine, adolescent medicine, and dual internal medicine/pediatrics physicians.^54,57–59^ Importantly, Gregory et al. highlight that these models have limited application for mothers of the 80% of U.S. infants who receive primary care from pediatricians.^56^ Thus, leveraging pediatric care access points for postpartum and interpregnancy care require coordination across multiple settings and provider specialties, spanning pediatrics, neonatology, internal medicine, family medicine, and obstetrics and gynecology.^56^

Clinicians interviewed in the Gregory et al. qualitative study articulated multiple structural, systemic, and policy barriers to multispecialty, multisetting dyadic care.^56^ These barriers included inadequate care handoffs between specialties, communication barriers across health care settings, lack of care coordination between specialties, inconsistent access to counterparts in other specialties during clinical urgencies, privacy restrictions limiting communication between maternal and infant care teams, scheduling systems created to accommodate individuals instead of dyads, and lack of comprehensive electronic health record (EHR) access to both mother and infant charts.^56^

Clinicians in the Gregory et al. study also articulated concerns about pitfalls that could potentially arise from delivering care outside of one's area of licensure and expertise and described that responsibilities for maternal follow-up care across specialties were poorly delineated.^56^ Gregory et al. also noted that while their study recruited clinicians involved in the care of preterm infants, clinician-reported barriers were not restricted to dyads who experienced PTB.^56^

Multispecialty mother/infant dyadic care, leveraging pediatric well-child visit access points, may provide compelling opportunities to address postpartum and interpregnancy care gaps, including after PTB. Gregory et al. report many structural, policy, and practice barriers to implementation of this dyadic model for longitudinal interpregnancy care delivery,^56^ while also proposing some practical steps to facilitate collaborative care models. These include repurposing existing EHR team-based communication for handoffs, establishing clinician directories and call schedules to ease cross-dyad consultations, and clinically colocating dyad relevant medical specialties.^56^

Beyond these local interventions, these data can inform roadmaps guiding the policy changes and quality initiatives required to promote routine multispecialty interpregnancy dyadic care. First, incorporating longitudinal postpartum care, including primary care transition, into perinatal Medicaid programmatic, policy, and quality transformations may incentivize and resource the health system collaborative infrastructures needed for implementation of multispecialty dyadic programs.^60^ Prior transformations effected by Centers of Medicare and Medicaid Services (CMS) and select state Medicaid agencies have successfully improved obstetric postpartum visit attendance and quality.^60^ Broad adoption of postpartum Medicaid extension, with implementation or planned implementation in 46 states, provides national opportunities to apply similar approaches to incentivize optimal care after PTB and other high-risk pregnancies.^61^

Second, CMS can require that health systems with public-facing hospital designations (*e.g.*, “Birthing-Friendly” Hospital Designations) transition patients to established multidisciplinary interpregnancy care after PTB or high-risk pregnancy. The “Birthing-Friendly” Hospital Designation was established in 2022 to help patients identify hospitals that have implemented maternal care best practices.^62^ CMS currently assigns “Birthing-Friendly” Hospital Designation to systems that participate in state or national pregnancy quality collaboratives and act on their recommendations.^63^

There remain many opportunities to iteratively expand designation requirements over time to include transition to multidisciplinary, longitudinal postpartum care. Third, coalitions, such as the Alliance for Innovation in Maternal Health, can set priorities and establish consensus bundles to support state agencies and health systems in implementing the complex cross-sector strategies needed to ensure high-quality, evidence-based postpartum and interpregnancy care, especially after complex pregnancy.^64,65^ These interventions will help to overcome the barriers and facilitate the solutions proposed by Gregory et al.^56^ to improve postpartum maternal health outcomes through systematization of dyadic interpregnancy care after complex pregnancy, including PTB.

## Abbreviations Used

ACOG: American College of Obstetrics and Gynecology
CMS: Centers of Medicare and Medicaid Services
EHR: electronic health record
IM: infant mortality
PRM: pregnancy-related mortality
PTB: pregnancy is preterm birth
